# Concurrent Diagnosis of Chronic Myeloid Leukemia and Follicular Lymphoma: An Unreported Presentation

**DOI:** 10.1155/2018/7493601

**Published:** 2018-09-09

**Authors:** Amy G. Starr, Sushma R. Jonna, Joeffrey J. Chahine, Bhaskar V. Kallakury, Chaitra S. Ujjani

**Affiliations:** ^1^MedStar Georgetown University Hospital, Department of Pathology, 3800 Reservoir Rd NW, Medical Dental Building, SE 200, Washington, DC 20007, USA; ^2^Lombardi Comprehensive Cancer Center, Department of Hematology and Oncology, MedStar Georgetown University Hospital, 3800 Reservoir Rd. NW, Washington, DC 20007, USA

## Abstract

Lymphadenopathy in chronic myeloid leukemia (CML) is usually due to extramedullary involvement with accelerated or blast phases of the disease. The occurrence of non-Hodgkin lymphoma (NHL) as a synchronous malignancy with CML is rare. We report a case of a 73-year-old male who presented with dyspnea and right-sided lower extremity edema in the setting of leukocytosis. Bone marrow evaluation indicated a chronic phase chronic myeloid leukemia (CML), confirmed by molecular testing. Imaging of the chest for persistent dyspnea revealed supraclavicular and mediastinal lymphadenopathy. Biopsy of the cervical node showed expanded lymphoid follicles with atypical germinal centers that were positive for CD10, BCL-2, and BCL-6, consistent with follicular lymphoma (FL). Nodal PCR demonstrated clonal IGH and IGK gene rearrangements, and FISH analysis was positive for IGH-BCL-2 fusion. Together, these tests supported the diagnosis of FL. Additionally, the lymph node showed paracortical expansion by maturing pan-hematopoietic elements, no blastic groups, and positive RT-PCR analysis for BCR-ABL1, indicating concomitant involvement by chronic phase-CML. To our knowledge, this is the first reported case of a patient with a concurrent diagnosis of CML and FL.

## 1. Introduction

The diagnosis of synchronous myeloid and lymphoid malignancies is a rare occurrence. The most commonly reported combination is of Philadelphia chromosome-negative myeloproliferative neoplasms (MPNs) and chronic lymphocytic leukemia (CLL) [1]. Laurenti et al. published the largest case series (*n*=46), finding that patients typically initially present with CLL and then subsequently develop an MPN [2]. The occurrence of chronic myeloid leukemia (CML) with a lymphoid malignancy is uncommon but has been noted with various non-Hodgkin lymphoma (NHL) histologies [3–6]. To our knowledge, this is the first reported case of a patient with a concomitant diagnosis of CML and follicular lymphoma (FL).

## 2. Case

A 73-year-old Caucasian male presented with three weeks of dyspnea, headache, and lower extremity edema. Initial labs were significant for marked leukocytosis with increase in myeloid precursors and rare blasts: white blood cell (WBC) 156 k/*μ*L, neutrophils 103 k/*μ*L, monocytes 7.1 k/*μ*L, eosinophils 1.6 k/*μ*L, basophils 0, and blasts 12 k/*μ*L. Other cell lines were normal with hemoglobin of 12.6 gm/dL and platelets of 242 k/*μ*L. Uric acid was elevated at 9.0 ml/dL, and lactate dehydrogenase was 860 units/L.

A bone marrow biopsy was performed and revealed a chronic myeloproliferative neoplasm. H&E stained slides of the core showed marked hypercellularity (99%) with profound myeloid hyperplasia and complete maturation to segmented neutrophils. Immature myeloid cells of all stages were appropriately present, without dysplasia or increased blasts. A moderate amount of reticulin fibrosis was seen. Giemsa stain of the aspirate confirmed the biopsy findings with blast count of less than 5%. By flow cytometric analysis, myeloid cells in the blast gate expressing CD34 accounted for less than 1% of total cells. Molecular diagnostic testing of the aspirate indicated the presence of the BCL-ABL1 p210-type transcript by RT-PCR with an international scale-normalized (ISN) copy number of 35.27%. Fluorescence in situ hybridization (FISH) testing for BCR-ABL1 fusion was present in 89% of cells. Together, these findings were consistent with a diagnosis of chronic phase-CML.

Given the symptoms of intermittent dyspnea in the absence of anemia, the patient underwent further evaluation. Stress echocardiogram indicated a normal left ventricular ejection fraction. Computed tomography (CT) chest with contrast enhancement revealed mediastinal, cervical, and supraclavicular adenopathy, without evidence of pulmonary embolism.

**Figure 1:**
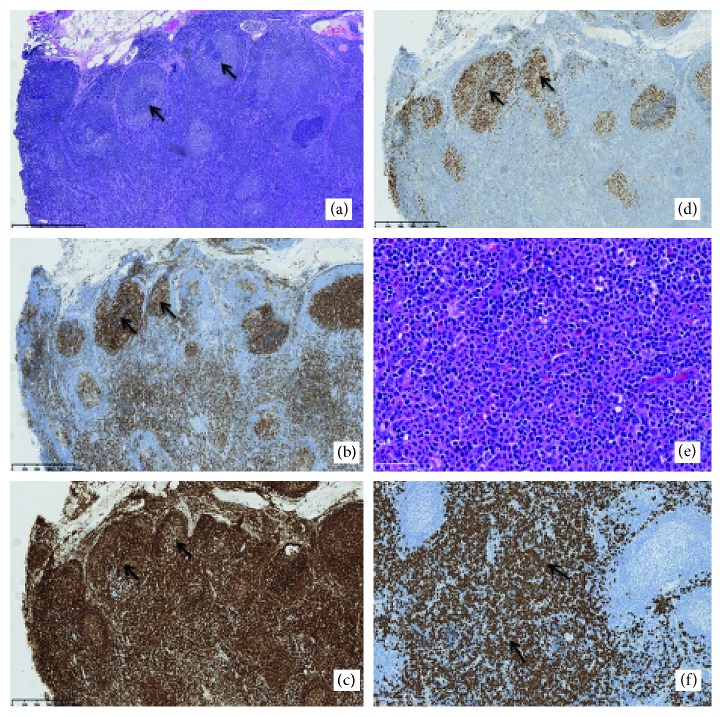
Lymph node histology and immunohistochemistry. (a) H&E section of lymph node showing atypical follicles (arrow). (b) CD10 immunostain highlighting FL cells within atypical germinal centers (arrow). (c) BCL-2 immunostain highlighting FL cells (arrow). (d) BCL-6 immunostain highligting FL cells (arrow). (e) Higher magnification of the area of paracortical expansion by neoplastic CML cells. (f) CD15 highlighting neoplastic CML cells (arrow). (a–d) 10x objective magnification, (e) 40x objective magnification, and (f) 20x objective magnification.

The patient subsequently underwent an excisional biopsy of the cervical lymph node, which revealed involvement with CML and FL (Figure 1). Atypical follicles with abnormal germinal centers and paracortical expansion by maturing pan-hematopoietic elements without blastic groups were noted. Immunohistochemistry (IHC) analysis showed that the atypical germinal centers expressed the pan-B cell marker CD20 and the germinal center marker CD10. There was coexpression of BCL2 and BCL6. The findings were consistent with low-grade follicular lymphoma, with fewer than 15 centroblasts per high-power field. PCR analysis for immunoglobulin heavy chain and kappa light chain gene rearrangements indicated monoclonality. Fluorescence in situ hybridization detected the IGH-BCL-2 translocation confirming follicular lymphoma.

IHC and flow cytometric analysis of the paracortical pan-myeloid hyperplasia showed no evidence of increased myeloblasts. Qualitative RT-PCR of the node detected the presence of the CML-type Mbcr (p210) fusion protein, and quantitative RT-PCR showed an ISN copy number of BCR-ABL transcripts of 0.642%, confirming nodal involvement by chronic phase-CML. Given the unusual presentation of concomitant CML and FL, further testing was done to evaluate for other abnormalities. PD-L1 expression was low (10%) using the 22C3 clone (Dako), and EBV was negative both by IHC for the viral latent membrane protein 1 (LMP1) and by Epstein–Barr encoding region in situ hybridization (EBER).

## 3. Clinical Course

The patient was initiated on imatinib for treatment of CML. It was chosen over the other available tyrosine kinase inhibitors due to drug interactions with his other medications. As he had no indications for treatment of the FL, he was clinically monitored with the watch-and-wait approach. Imatinib was well tolerated and only briefly held for thrombocytopenia. By three months of treatment, the WBC normalized to 5.1 k/*μ*L, and BCR-ABL1 by RT-PCR of peripheral blood decreased to an ISN value of 30%. His symptoms of dyspnea resolved. At 6 months, bone marrow biopsy was normocellular, consistent with complete morphologic remission. Flow cytometric analysis confirmed the absence of increased myeloblasts or involvement by FL. He achieved a partial cytogenetic remission, as karyotyping indicated persistence of Philadelphia chromosome in 15% of cells. BCR-ABL1 by RT-PCR of peripheral blood showed an ISN value of 1.15%. Repeat PET scan to reassess lymphadenopathy showed stable disease. He is currently continuing on imatinib for CML and is being followed clinically for FL.

## 4. Discussion

The diagnosis of both myeloid and lymphoid neoplasms in a single patient, whether simultaneous or sequential, is extremely rare, with an overall incidence of less than 1% [1]. The majority of cases (66%) have sequential presentations while only 34% present concurrently [2]. Here, we present the third known case of a patient with nodal involvement by both FL and chronic phase-CML. Both previously documented cases occurred in patients with CML who developed follicular lymphoma after initial diagnosis [6, 7].

Extramedullary disease (EMD) in CML, including nodal involvement, typically occurs in the accelerated phase (AP) or blast phase (BP), which accounts for 15% of new CML diagnoses [8, 9]. The most common sites of extramedullary involvement are bone, lymph nodes, skin, soft tissues, and the central nervous system [10]. In a study by Inverardi et al., half of the patients were in chronic phase (CP) CML at the time EMD was diagnosed, and the other half were in AP or BP. Those patients that were in CP-CML progressed to BP CML within 4 months, implying that extramedullary disease may herald impending blast crisis even if blast transformation was not present initially [10]. These early studies evaluated patients before the benefit of tyrosine kinase inhibitors (TKIs) was well understood. The clinical course of patients with CML has evolved since the advent of TKIs, which have significantly impacted the prognosis of the disease. In spite of this, physicians should closely monitor patients with evidence of EMD, as they may be at higher risk for blast formation. In the present case, the patient's lymphadenopathy was concerning for EMD leading to a lymph node biopsy. Evaluation for AP and BP was confounded by the unexpected finding of FL. Further analysis by molecular testing confirmed lymph node involvement by CP-CML without any increase in blast cells.

Postulations regarding pathogenesis for the dual presentations of myeloid and lymphoid malignancies include genetic instability, specific chemotherapy drugs, radiotherapy, and environmental exposures as predisposing factors. Genetic mutations of oncogenes, such as those of the RAS family, or tumor suppressor genes, such as p53, are noted in patients with CML and NHL [11–14] and theoretically predispose to multiple malignancies. In a recent study, data from the randomized control trial, CML study IV, were analyzed to evaluate the impact of long-term use of TKIs in the development of secondary malignancies. Patients with CML on TKIs had a significantly higher standard incidence ratio of 3.33 in males and 4.29 in females for development of NHL compared with a matched German population. The median time from diagnosis of CML to the diagnosis of another malignancy was 2.4 years [15]. The effect of TKIs on DNA repair mechanisms is thought to be a potential mechanism for this finding based on preclinical studies [16]. In a recently published case report, a patient experiencing a complete cytogenetic response with imatinib for CML developed FL three years after initiation of the first generation TKI. He received rituximab monotherapy for the FL, resulting in a partial remission. Although he continued to receive imatinib, eventually achieving a major molecular remission, he subsequently lost this deeper response after another three years, followed by a progression of FL. Treatment with a second-generation TKI resulted not only in a major molecular remission of the CML, but also a complete remission for the FL [7]. As our patient had concurrent nodal involvement by CP-CML and FL at initial presentation, the presence of these two malignancies may represent independent events.

Pathologists and oncologists must be aware of the possibility of concurrent hematologic malignancies. Lymphadenopathy in a patient with CML may represent blast crisis, but a distinct lymphoid malignancy is also possible. When this is suspected on morphologic examination, further evaluation using ancillary techniques including immunohistochemistry, flow cytometry, and PCR-based assays offer conclusive and accurate diagnosis. As the management of each of the synchronous malignancies often differs, this distinction is important for clinicians to make treatment decisions. Future investigations to evaluate the pathogenesis of the dual occurrence of myeloid and lymphoid malignancies are warranted to better understand and manage patients.
